# Factors associated with private health facilities reporting malaria in the national health management information system in Zambia: a cross sectional study

**DOI:** 10.11604/pamj.2020.37.203.18829

**Published:** 2020-10-29

**Authors:** Angela Gama, Ignatius Banda, Fred Kapaya, Mercy Mwanza Ingwe, Japhet Chiwaula, Anthony Yeta, Elizabeth Chizema Kawesha, Busiku Hamainza, Gershom Chongwe

**Affiliations:** 1 Zambia Field Epidemiology Training Programme (ZFETP), Ministry of Health, Lusaka, Zambia,; 2 Zambia National Public Health Institute, Lusaka, Zambia,; 3 Zambia National Malaria Elimination Centre, Lusaka, Zambia,; 4 University of Zambia, Lusaka, Zambia

**Keywords:** Malaria, surveillance, health management information system, private health facilities, Zambia

## Abstract

**Introduction:**

Zambia has moved from accelerated malaria burden reduction to malaria elimination which requires the national malaria surveillance system to capture all cases from both the public and private sector. This study investigated challenges and factors associated with private health facilities reporting malaria in the national health management information system (HMIS).

**Methods:**

a structured questionnaire was administered to the heads of 139 private health facilities in three provinces where approximately 85% of private health facilities are found in Zambia. Logistic regression was performed, and the outcome variable was reporting malaria in the HMIS. Epi Info® version 7 was used to conduct multivariable logistic regression to determine factors associated with private facilities reporting malaria in HMIS.

**Results:**

private health facilities that had been operating for more than 20 years had three (3) times increased odds of reporting malaria in HMIS (AOR = 3.22, 95% CI: 1.23, 8.42; P-value = 0.02) compared to those that had been operating for less than 20 years. The private facilities that had staff who were aware about malaria surveillance (AOR = 2.06 95% CI: 1.38, 3.99, P-value = 0.01) had two times greater odds to report malaria in HMIS compared to those that were not aware. Lack of information and training in surveillance was identified as the main barrier for private facilities to report malaria in HMIS.

**Conclusion:**

as Zambia progresses towards malaria elimination, there is need to increase awareness and training of private providers on malaria surveillance to improve reporting in HMIS.

## Introduction

Surveillance is core in malaria control and elimination. However, the World Health Organisation (WHO) estimates that only 19% of the 214 million cases that occurred globally in 2015 were detected and reported through national malaria surveillance systems [1]. One of the contributing factors was that malaria surveillance data from private health facilities were not being captured in routine surveillance [2]. In many countries, the private sector has a key role in providing malaria care; It is estimated that approximately 40% of patients with fever seek care in the private sector and with 35% of febrile children in sub-Saharan Africa being treated by private providers [3]. Despite the private sector providing malaria services, most National Malaria Control Programmes routinely collect malaria data for surveillance mainly from government health facilities and this is the main dataset that is reported to the World Health Organization [4,5]. This incomplete reporting of malaria incidence can result in a very inaccurate picture of the distribution of malaria and underestimation of the disease burden for the National Malaria Control Programme.

Many countries and regions worldwide, including Zambia, have in the past few years pledged to eliminate malaria, and this requires commitment from both government and private sectors [1]. In order for malaria elimination to be successful, it is important to have strong surveillance systems capable of giving an accurate picture of malaria incidence over time and place [6]. In the current Zambia National Malaria Elimination Strategic Plan 2017 to 2021, the major goal is to eliminate local malaria infection and disease by 2021 and surveillance is one of the key interventions [7]. The strategic plan is premised on the use of epidemiological data to direct program implementation which will require the national malaria surveillance system to capture all the cases both from the public and private sector to ensure a complete and accurate picture of malaria incidence.

Malaria is a notifiable disease in Zambia [8] and therefore private facilities are required to report cases to the national HMIS. The HMIS serves as the principal health care monitoring system for collecting routine surveillance information in Zambia. Information in the HMIS has been useful for track broader trends in malaria and direct program implementation in Zambia. As Zambia pursues malaria elimination, enhanced surveillance systems with the involvement of the private sector is increasingly critical. In addition, engaging with the private sector in surveillance is an opportunity to grow the country´s evidence base. We carried out a survey to determine the number of private facilities that reported malaria and understand the challenges and factors associated with private health facilities reporting malaria in the national HMIS.

## Methods

**Study design and sampling strategy:** a list of health facilities obtained from the Health Professions Council of Zambia (HPCZ), the body responsible for the licensing and regulation of all private health facilities in the country, was used as a sampling frame [9]. There was a total of 546 private health facilities registered with HPCZ. A cross-sectional survey was done in Lusaka, Southern and Copperbelt provinces, where about 466 (85%) of all private healthcare facilities in Zambia are located. Out of a total of 546 facilities on the list, 266 were in Lusaka, 164 in the Copperbelt and 36 from Southern province. All the facilities that offered malaria services regardless of their size were included. Facilities such as dental clinics and optician clinics were excluded. After this process, the remaining private facilities were 304, of which 179 were from Lusaka province, 99 from the Copperbelt province and 26 were from Southern province. Information on malaria reporting rates by private facilities in Southern, Copperbelt and Lusaka province of Zambia was extracted from the HMIS. To determine challenges and factors associated with reporting malaria to the HMIS by private health facilities in Southern, Copperbelt and Lusaka province, a sample size was determined using Open Epi®. Assumptions for the sample size was a margin of error of five percent, Confidence level of 95%, a design effect of one and an expected frequency of 21% [1]. Design effect of one was used for sample size calculation because of the stratification by province hence reducing the variability of results. Therefore, the variation was assumed to be like simple random selection. Based on these assumptions, sample size for the study was estimated to be 139 private health facilities. Probability proportion to size sampling was used to estimate the sample size of private health facilities in each province, which resulted in eighty-two participants for Lusaka province, forty-five for Copperbelt and twelve for Southern province. Random sampling in Microsoft Excel was done by creating a random number for each private health facility.

**Data collection plan and tools:** a private health facility was defined as any outlet or, facility that provides clinical or diagnostic services and is not managed by a national or local government authority [10]. Information on the number of private facilities that reported malaria was collected from the national health management information system (HMIS) in the Copperbelt, Lusaka and Southern province of Zambia from 2012 to 2017. A structured questionnaire was used to collect information from the heads of private health facilities (or individuals nominated by the heads of the facilities) on challenges and factors associated with private facilities participating in malaria surveillance. The questionnaire was piloted before it is administered. Since reporting is paper based in Zambia, private health facilities enter their monthly malaria data in the HMIS data collection tools which are submitted to the district health office. The questionnaire had questions on whether the private health facility reports malaria in the HMIS. The private health facilities that reported malaria provided copies of the reports submitted to the district health office. Therefore, reporting malaria in HMIS in the study was defined as a private facility that submitted their monthly reports to the district health office. The questionnaire also included questions on the number of years the facility has been operating; if the facility had, someone trained in malaria surveillance and the respondent being aware about malaria surveillance.

**Data analysis:** descriptive statistics were used to determine the frequencies of private health facilities reporting malaria in HMIS. Cross tabulation was done to determine the distribution of factors that were associated with private facilities reporting malaria in the national HMIS. This was followed by unadjusted logistic regression. Significance at unadjusted logistic regression was set at a p-value of 0.1 and a 95 percent confidence interval. Odds ratios with their 95% confidence intervals (CI) were calculated to compare reporting private health facilities with those that did not report. Variables that were found to be significant at unadjusted logistic regression were then fitted into the multivariable logistic regression to control for confounding and to develop the final model of factors associated with private facilities reporting malaria in the HMIS. P-value < 0.20 was used to select variables for inclusion into the initial multiple logistic regression model. The final model was developed by investigator-led backwards elimination, dropping the least significant independent variable until all the remaining predictor variables were significant (p-value < 0.05).

**Ethics approval and consent to participate:** ethics approval (Reference number 2017-Jul-029) was obtained from the Excellence in Research Ethics and Science (ERES) Converge Research Ethics Committee. Authority to conduct the study was obtained from the Zambia National Health Research Authority. Permission was sought from the Ministry of Health to use the HMIS data. Informed consent was obtained from the heads of the selected private health facilities before administering the questionnaire. Confidentiality was guaranteed by using identifiers for the sampled private health facilities. People involved in data collection were oriented on how to handle data and ensure confidentiality. Collected data was secured and used only for this study.

## Results

**Malaria reporting in the HMIS:** information on malaria cases from private facilities was collected from the HMIS from 2012 to 2017. There were a total 99, 13 and 27 private facilities in the HMIS in the Copperbelt, Southern, and Lusaka province respectively from 2012 to 2017. In 2017, 36% (n = 36/99) of the private health facilities in the Copperbelt, 15% (n = 4/27) in Lusaka and 8% (n = 1/13) in Southern province reported malaria in the HMIS (Table 1). Monthly reporting rate was less than 50% in the three provinces (Table 1).

**Table 1:** private facilities in the HMIS that reported malaria in Copperbelt, Lusaka and Southern provinces, in Zambia, 2012-2017, (n = 139)

Year	Total No. of private health facilities in the HMIS	No. of private health facilities that reported in the HMIS (%) a	Total number of expected reports in the HMIS b	Total number of reports submitted in the HMIS (%) c
**Copper belt**				
2012	99	44 (44)	1188	459 (39)
2013	99	43 (43)	1188	456 (38)
2014	99	43 (43)	1188	304 (26)
2015	99	36 (36)	1188	558 (47)
2016	99	36 (36)	1188	539 (45)
2017	99	36 (36)	1188	322 (27)
**Lusaka**				
2012	27	1 (4)	324	3 (1)
2013	27	2 (7)	324	18 (6)
2014	27	5 (19)	324	63 (19)
2015	27	6 (22)	324	57 (18)
2016	27	3 (11)	324	28 (9)
2017	27	4 (15)	324	47 (15)
**Southern**				
2012	13	2 (15)	156	16 (10)
2013	13	2 (15)	156	18 (12)
2014	13	2 (15)	156	12 (8)
2015	13	3 (23)	156	17 (11)
2016	13	2 (15)	156	44 (28)
2017	13	1 (8)	156	12 (8)

(%)a = No. of private health facilities that reported in the HMIS/total private facilities in the HMIS; Total number of expected reports in the HMIS b: total number of health facilities in the HMIS x 12 months; Reporting rate- (%)c = total number of reports submitted in the HMIS /total number of expected reports in the HMIS

**Factors associated with private facilities reporting malaria in the HMIS:** out of the 139 private facilities visited, 45 (32%) were from the Copperbelt, 82 (58%) were from Lusaka and 12 (9%) were from Southern province (Table 2). Ninety percent (n = 125/139) of the private facilities were privately owned, eight percent (n = 11/139) were owned by faith-based institutions and three percent (n = 2/139) were owned by parastatal (or semi-autonomous, government-supported) institutions. Fifty percent (n = 70/139) of the private health facilities had been operating for 1-10 years. Sixty-seven percent (n = 93/139) of the private health facilities had laboratories and 75% (n = 104/139) had computers. Most of the private health facilities had less than five nurses (n = 103/139, 74%) and medical doctors (n = 125/139, 90%). A few (n = 12/139, 9%) private facilities visited had personnel trained in malaria surveillance. Fifty-four percent (n = 79/139) of the respondents were aware of malaria surveillance. Most (n = 124/139, 89%) of the respondents agreed that it is important for private health facilities to report the malaria cases in the HMIS. Lack of information and training in surveillance was the most (n = 74/139, 53%) common challenge that most private facilities had in reporting malaria data in the national surveillance system (Table 3).

**Table 2:** characteristics of private health facilities in Copperbelt, Lusaka and Southern provinces, in Zambia (n = 139)

Characteristic	Report Malaria in HMIS [n (%)]		
	Yes (n = 60)	No (n = 79)	Total (n = 139)
**Province**			
Copperbelt	32 (53)	13 (16)	45 (32)
Lusaka	26 (43)	56 (71)	82 (58)
Southern	2 (3)	10 (13)	12 (9)
**Type of facility**			
Privately owned	51 (85)	74 (94)	125 (90)
Faith based	7 (12)	4 (5)	11 (8)
Parastatal	2 (3)	1 (1)	3 (2)
**Number of years the facility has been operating**			
1-10	20 (33)	50 (63)	70 (50)
11-20	10 (17)	13 (16)	23 (17)
≥21	30 (50)	16 (20)	46 (33)
Median (IQR)			10 (5-22)
**Number of facility with logistics and equipment**			
Electricity backup (n = 139)	48 (80)	48 (61)	96 (69)
Laboratory (n = 139)	41 (68)	52 (66)	93 (67)
Internet (n = 139)	37 (64)	49 (62)	86 (63)
Computer (n = 139)	46 (77)	58 (73)	104 (75)
**Number of nurses** [Median (IQR)			3 (1,5)
<5	31 (52)	72 (91)	[103 (74)]
≥5	29 (48)	7 (9)	26 (26)
**Number of medical doctors** [Median (IQR)]			[1 (1,2)]
<5	51 (85)	74 (94)	125 (90)
5-9	9 (15)	5 (6)	14 (10)
**Number of record clerk** [Median (IQR)]			[1 (0,2)]
<5	53 (88)	76 (96)	129 (93)
5-9	7 (12)	3(4)	10 (7)
**Malaria surveillance**			
Number of private facilities with trained personnel in surveillance	9 (15)	3 (4)	12 (9)
Respondents that were aware of malaria surveillance	43 (72)	32 (41)	75 (54)
Important for private health facilities to report malaria in HMIS	51 (85)	73 (92)	124 (89)

IQR: interquartile range

**Table 3:** challenges private facilities have to report malaria in HMIS in Copperbelt, Lusaka and Southern provinces, in Zambia (n = 139)

Characteristic	Reporting malaria in HMIS [n (%)]		
Challenges	Yes (n = 60)	No (n = 79)	Total (n = 139)
Lack of information and training in surveillance	23 (38)	51 (65)	74 (53)
Poor coordination between government private sector	11 (18)	18 (23)	29 (21)
Lack of clear guidelines for submitting data (no tools)	6 (10)	19 (24)	25 (18)
Lack of time	7 (12)	9 (11)	16 (12)
Private facilities are not given ITN^*^ for maternity and under 5years	5 (8)	10 (13)	15 (11)
No transport to take reports (reporting is paper based)	1 (2)	14 (18)	15 (11)
Lack of acknowledgment of efforts	2 (3)	8 (10)	10 (7)
Inadequate human resource	0 (0)	5 (5)	5 (3)
No feedback is provided after submitting reports	3 (4)	0 (0)	3 (2)
Poor record keeping in private facilities	0 (0)	1 (1)	1 (1)

* ITN = Insecticide-treated mosquito net

**Factors associated with private facilities reporting malaria in the HMIS:** in multiple variable logistic regression analysis, after adjusting for the confounding effects of the number of record clerks and doctors the private health facility had and having electricity back up, private facilities that had been operating for more than 20 years had three times increased odds of reporting malaria in HMIS (AOR = 3.22, 95% CI: 1.23, 8.42; P-value 0.02) compared to those that had been operating for less than 20 years. Private facilities that were in the Copperbelt province (AOR = 2.20 95% CI: 1.35, 3.58; P-value 0.01) had two times greater odds of reporting malaria in HMIS compared to those that were in Lusaka province. The private facilities that had staff who were aware about malaria surveillance (AOR = 2.06 95% CI: 1.38, 3.99; P-value 0.01) had two times greater odds of reporting malaria in HMIS compared to those that were not aware (Table 4).

**Table 4:** factors associated with private health facilities reporting malaria in the HMIS in Copperbelt, Lusaka and Southern provinces, in Zambia (n = 139)

Factors	UOR(95% CI)	P-Value	AOR(95%CI)	P-Value
**Number of years the facility has been operating**				
1-10	1		1	
11-20	2.93 (1.26, 6.77)	0.01	0.74 (0.19, 2.81)	0.66
≥21	5.57 (2.71, 11.44)	<0.01	3.22 (1.23, 8.42)	0.02
**Province**				
Lusaka province	1		1	
Southern province	0.76 (0.22, 2.60)	0.67	0.43 (0.08, 2.43)	0.33
Copperbelt province	4.58 (2.35, 8.92)	<0.01	2.20 (1.35, 3.58)	<0.01
**Number of doctor**				
<5	1		1	
5-10	3.4 (1.16, 10.07)	0.025	1.10 (0.29. 4.18)	0.88
**Number of nurses**				
<5	1		1	
≥5	7.80 (3.56, 17.11)	<0.01	4.92 (2.03, 11.93)	<0.01
**Has any of your staff been trained in surveillance**				
No	1		1	
Yes	8.10 (2.28, 28.79)	<0.01	4.34 (1.00, 18.85)	0.05
**Aware about malaria surveillance**				
No	1		1	
Yes	17.34 (8.18, 36.78)	<0.01	2.06 (1.38, 3.99)	0.01

UOR = unadjusted odds ratio; AOR = adjusted odds ratio; CI = confidence interval

## Discussion

Private facilities provide care for malaria patients and therefore, are an important source of surveillance malaria data. However, this study revealed that very few private facilities reported on malaria in the HMIS in the sampled three provinces. These findings are consistent with those reported in Kenya, Uganda, South Africa, and other countries that also receive HMIS data from the private sector [11-13]. In South Africa, studies have shown that only 26% of malaria cases diagnosed in the private sector are being reported [13]. Epidemiological information is used to make decisions on interventions in Zambia, therefore, low reporting rates of malaria by private facilities in the HMIS may result in underestimation of the actual malaria burden which may result in deployment of inappropriate interventions. Different strategies have been devised to improve private facilities reporting on malaria. For instance, in Ghana, private health facilities such as hospitals and clinics are supervised by the district health directorate. In addition, the Ghanaian National Malaria Control Program sends data managers to provide support on data related issues to both public and private facilities [5].

The study findings indicate that few private facilities had someone trained in malaria surveillance with half of the respondents from private facilities not being aware about malaria surveillance and the HMIS. Lack of information, training in surveillance and clear guidelines and tools for submitting data was identified by most respondents from private facilities as a major challenge that private facilities have in reporting malaria in the HMIS. Considering this result, it can be concluded that the lack of training of private healthcare providers in malaria surveillance is an important reason for under-reporting of malaria in the HMIS. Training of private sector providers has been shown to improve adherence to national guidelines for anti-malarial prescription and may be one of the most operationally achievable and cost-effective way to improve malaria case management and surveillance in the private sector [14-17]. Combining training with other interventions that reinforce each other are likely the most effective approach [18], and integrated training with social marketing approaches, referral systems and increased local regulatory oversight [19].

Another important challenge reported by private facilities was poor coordination between the government and the private sector. One of the factors contributing to the poor coordination between the government and private sector is because in most countries, the national malaria programmes are established by the public sector, therefore the trainings are provided to the public providers with little or no involvement of the private providers [15]. As a result, trainings usually target the public sector only and the private sector are excluded. According to Global Fund [5] technical updates on malaria emphasize the importance of involving the private sector to ensure effective malaria case management for all patients, as well as accurate malaria surveillance. As Zambia approaches malaria elimination, it is essential that the National Malaria Elimination Centre (NMEC) facilitates linkages and routine interaction between the public and private providers. Linkages can be established through regular shared trainings, meetings at provincial or district levels so that private providers feel that they are part of the elimination efforts [15].

Human resource is also an important factor that affects reporting. For instance, the study found that private facilities that had more than five nurses had five times greater odds of reporting malaria in the HMIS compared to those than had less than five nurses. In most private facilities the responsibility of reporting in the HMIS was assigned to the chief nursing officer. Thus, the nursing officers in private facilities that had more than five nurses might have had more time to prepare the reports compared to those that had fewer nurses. Private facilities that were in the Copperbelt province had a two times higher odds of reporting malaria in the HMIS compared to those in Lusaka province. This could be due to the different systems in the three provinces. It was found that the private facilities in the Copperbelt directly report in the HMIS while for Lusaka province most private facilities reported their data to the public facility in the catchment area near their location. It is easier to monitor the private facilities that are reporting and the number of malaria cases when private facilities report directly in the HMIS.

Being aware about malaria surveillance was associated with the private health facility reporting data to the HMIS. In addition, having staff trained in surveillance was also associated with reporting. Several other studies have also reported strong associations between trained staff in surveillance and reporting [15,20,21]. Therefore, a great potential improvement in private sector reporting malaria in the HMIS might be achieved through training which can provide private providers with information, updates on guidelines on malaria case management, surveillance and elimination effort and can act as a platform to discuss the challenges they may face. Data were collected from private health facilities in three out of ten provinces in Zambia which is a limitation of this study. Despite this limitation, it is worth noting that 85% of the country´s private health facilities are found in the three provinces. The importance of these findings lies in their implications for improving malaria surveillance as the study showed challenges and factors that are associated with private facilities reporting malaria in the HMIS. One of the important findings in the study was that most respondents from private facilities agreed that it is important for private health facilities to report malaria data in the surveillance system. This finding may suggest willingness of the private sector to participate in the malaria surveillance system.

**Implications for public health:** the study has shown that few private facilities reported malaria in HMIS, suggesting that previously reported data from the HMIS might have been underestimating the disease burden. As Zambia is approaching malaria elimination, it is essential that each malaria case is captured in the national surveillance system. There is a need to strengthen the involvement of the private sector in the overall national surveillance system. The factors associated with private facilities reporting malaria in the HMIS found in this study emphasizes the need to focus on ensuring awareness and frequent trainings of private providers, on malaria surveillance. Therefore, strengthening training on surveillance and reporting systems in the training schools of health workers so that as they graduate this could already be inculcated in their practice could be one way of improving disease surveillance and reporting. Trainings of the private sector providers on malaria case management, surveillance and elimination efforts is important in the process of malaria elimination in Zambia, however the private sector may not make themselves available for engagement due to “loss” of business when they are away at a training, therefore, the findings also suggest the need for further research to understand how to effectively involve the private sector in malaria elimination efforts in Zambia.

## Conclusion

The study has demonstrated that very few private facilities reported malaria in HMIS. The main factors associated with private health facilities reporting malaria in HMIS included the private health facility operating more than 20 years, the staff being aware of and trained in malaria surveillance and the private health facility having more than five nurses. Lack of information and training in malaria surveillance was identified as the main barrier for private facilities to report malaria in HMIS. As Zambia progresses towards malaria elimination, working with the private sector is important to ensure people seeking treatment from private provider´s access effective case management and private providers report all the malaria cases in the national surveillance system.

### What is known about this topic

- The private sector contributes to providing malaria services;
- Few private health facilities report their malaria data to the national surveillance system.

### What this study adds

- Few private health facilities reported malaria in the HMIS in three provinces where most of the private health facilities are found in Zambia;
- Health staff being aware of and trained in malaria surveillance and having more than five nurses in the private health facility was associated with reporting malaria in HMIS;
- Lack of training and information on malaria surveillance was identified as the main challenge for private facilities to report malaria in HMIS.
